# Alien Algae Species Invasions in Humic Rivers within Weakly Human Impact Basin

**DOI:** 10.3390/life14010061

**Published:** 2023-12-29

**Authors:** Pavel Kulizin, Ekaterina Vodeneeva, Nikita Martynenko, Ekaterina Sharagina, Alexander Okhapkin

**Affiliations:** 1 Laboratory of Water Ecosystems, Institute of Biology and Biomedicine, Lobachevsky State University of Nizhny Novgorod, 23 Gagarin Avenue, Nizhny Novgorod 603022, Russia; vodeneeva@mail.ru (E.V.); ajugareptans@mail.ru (E.S.);; 2 A.N. Severtsov Institute of Ecology and Evolution, Russian Academy of Sciences, 33 Leninsky Avenue, Moscow 119071, Russia

**Keywords:** phytoplankton, raphydophytes, dinophlagellates, diatoms, taiga zone, phylogenetic analysis, invasions, the Vetluga river, the Kerzhenets river, the Vishnya river, the Cheboksary reservoir water basin

## Abstract

Increasing anthropogenic influence and climate change are leading to significant transformations in living conditions for indigenous representatives of aquatic communities. The problem of alien species invasions is actively discussed in the example of large rivers and water reservoirs, but medium and small rivers with weak anthropogenic influence have been insufficiently studied in this aspect. With the help of analysis of literary data and the results of our own long-term observations of phytoplankton using morphological and molecular genetic methods in some left-bank Volga tributaries, we identified six invasive species of different taxonomic groups of algae, with a predominance of diatoms. The relevance of using both traditional and modern approaches to identifying invasive algae species is revealed. Such invasive species as *Thalassiosira incerta*, *T. faurii*, *Skeletonema subsalsum*, *Unruhdinium kevei*, and *Gonyostomum semen* were part of planktonic communities; the benthic species *Plagiotropis lepidoptera* var. *proboscidea* sometimes reached a significant level of development (up 6 to 44% from total biomass) in plankton. It was demonstrated that some algae species have firmly taken the position of dominants and subdominants in planktonic algae communities. The expansion of alien representatives of microphytobenthos was noted in the Volga River basin for the first time. For *Gonyostomum semen*, its European origin was revealed, for plankton and benthic diatom—Ponto-Caspian. Our study showed that the processes of invasion and subsequent development of alien species take place in habitats with weak anthropogenic influence, which is likely determined by the hydrological, hydrochemical, and climatic changes in river basins and the high adaptive capabilities of invasive species.

## 1. Introduction

The problem of alien species invasions is one of the most important ones at present. The movement of living organisms within not only certain territories but also between countries and continents of the globe has occurred at all times. However, at the end of the 19th and the beginning of the 20th centuries, this process intensified significantly due to the increasing anthropogenic impact on the environment and global climate change [1].

Like other organisms, algae can be invasive, so they are able to actively invade habitats that are unusual for them, contributing to a decrease in biodiversity and changes in structural indicators in their habitat. Most publications [2,3] concerned with this problem were devoted to marine macroscopic alien algal invasions. Microscopic algae and the features of their introduction into fresh waters were studied to a lesser extent. Information about the invasive processes of microscopic forms of algae is provided for the water bodies of North America [4,5], Europe [1,2], and Asia [6]. The active introduction and adaptation of algae into freshwater bodies occur more intensively in the presence of spatiotemporal gradients in the content of minerals and nutrients. The main reasons for the emergence of new species are considered to be changes in climate, hydrology, and increased intensity of transport communications (with ballast water, e.g.,).

In recent years, invasive processes have significantly become more prominent in the basin of the large European river Volga. Since the formation of the Volga reservoir cascade, there has been a gradual transformation of hydrological and hydrochemical parameters, in particular, an increase in such parameters as mineralization, the content of alkali and alkaline earth metal ions, sulfates, chlorides, and, as a result, a rise of the trophic status of many water bodies in their basins [7,8]. These changes are important limiting factors at the initial stages of the spread of invasive species, including algae, since they reached their greatest development in the more mineralized waters of the Middle and Lower Volga [8,9]. Long-term studies (from the late 1960s to the present) made it possible to trace the dynamics of the invasive algae species composition and their productivity indicators and to predict the further expansion of new algae species, especially ones from the brackish-water group [10].

The purpose of the present work is to identify the invasive algae species composition with traditional and modern approaches and assess the degree of their development in the plankton of the left bank tributaries of the Volga River within its weak human impact basin.

## 2. Materials and Methods

### 2.1. Sample Area

**Figure 1:**
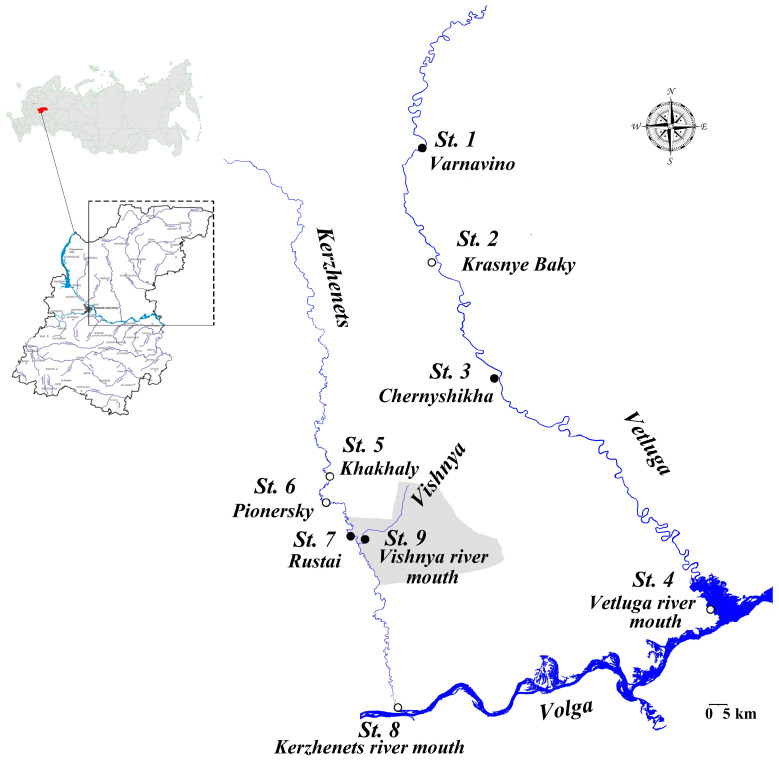
Investigation area with stations of water sampling. Stations 1–4 are on the Vetluga River; 5–8—on the Kerzhenets River. st. 1—nearby the Varnavino village (2010); st. 2—near the Krasnye Baky village; st. 3—near the Chernyshikha village; st. 4—the Vetluga River mouth; st. 5—near the Khakhaly village; st. 6—near the Pionersky village; st. 7—near the Rustai village; st. 8—the Kerzhenets River mouth; st. 9—the Vishnya River mouth. Area marked with gray color is the territory of the Kerzhensky State Nature Biosphere Reserve.

Phytoplankton samples from three morphologically different rivers of the Left Bank of the Volga River (Nizhny Novgorod region, Russia), such as the Vetluga (large), the Kerzhenets (middle) and the Vishnya (small one) rivers, were used as the material for the present work. Water sampling was carried out with the use of generally accepted methods [11,12,13] at water monitoring stations presented in Figure 1. The coordinates and the additional information about the sampling station are in Table 1. Phytoplankton samples were collected in the following years: 2002 (12 samples), 2008 (11 samples), 2010 (14 samples), 2014 (18 samples), 2016 (16 samples), 2018 (16 samples), 2019 (19 samples). Water samples for molecular genetic analysis were taken in 2021. In this work, we also used archival data from the Department of Botany and Zoology of Lobachevsky University (Nizhny Novgorod, Russia), including the results of sample analysis for the period 1975–1990, and permanent diatoms microslides.

### 2.2. Sample Collection, Morphological Identification, Hydrochemical and Statistical Analysis

We collected 0.5 L volume water samples from the surface layer of water and preserved them with a 4% iodine-formalin solution. Laboratory analysis of samples was carried out by the method of phytoplankton sedimentation with further filtration through fine-porous membrane filters “Vladipor” (Vladimir, Russia) with a disk diameter of 35 mm and a pore size of 2.5 microns. All the methods were described earlier in the work of Vodeneeva et al. [13].

Identification of algae species, size measurement and cell counting were carried out in a Najott’s chamber using the following light microscopes: MEIJI MT4000 series (Meiji Techno, Tokyo, Japan) at the Institute of Biology and Biomedicine of the Nizhny Novgorod State University (Nizhny Novgorod, Russia), Nexcope NE920 (Ningbo, China) with a BestScope_BUC5F-830CC camera (Beijing, China) at the Institute of Ecology and Evolution A.N. Severtsov of the Russian Academy of Sciences. The species composition of centric diatoms was studied with the use of a JSM-6510LV scanning electron microscope (Tokyo, Japan) at the Center for Shared Use of Electron Microscopy at the I.D. Papanin Institute of Biology of Inland Waters, RAS. We used modern keys and taxonomic summaries for algae species identification, all of which were given in previously published works by Vodeneeva et al. [13]. Changes in algae taxonomy when compiling the species list were verified using the international database AlgaeBase [14].

We performed a hydrochemical analysis on the base of a laboratory of the Shared Use Center of the Institute of Chemistry, Lobachevsky University, Nizhny Novgorod, Russia with the help of the following equipment: a V-1100 spectrophotometer (JP Selecta, Barcelona, Spain), a «CAPEL 105 M» capillary electrophoresis system (Saint-Petersburg, Russia), a PRODIGY inductively coupled plasma atomic emission spectrometer (ICP spectrometer) (Teledyne Leeman Labs., Hudson, NH, USA), and an HI 98130 water analyzer (HANNA Instruments, Salaj, Romania). The considered parameters were: iron, magnesium, calcium, chlorides, nitrites, sulfates, phosphates, bicarbonates, pH, water chromaticity, and total mineralization.

Spearman’s rank correlation coefficient (Rs) was used to study the relationship between abiotic environmental factors and quantitative rates of development of invasive species. We used the Wilcoxon test (W) for analyzing the significance of interannual differences in chemical parameters of the environment. We used Statistica 13 software for this.

### 2.3. DNA Extraction, Amplification, and Sequencing

For living cell isolations and for conducting molecular genetic research, the phytoplankton samples were collected in 2021. Due to the fact that the cultivation of studying organisms is a complicated process, we used single-cell polymerase chain reaction (scPCR) in the molecular analysis of isolates. Individual living cells were picked up with the glass micropipette. Each cell was washed in 6–8 droplets of sterile water to prevent contamination with foreign DNA and transferred into a 100 μL tube with a small amount of water to avoid drying. Further, this tube was frozen for cell wall destruction. Then, 30 μL of InstaGeneTM Matrix (BioRad, Hercules, CA, USA) was added to defrosted tubes, and the total DNA was extracted according to the manufacturer’s protocol.

The part of nuclear SSU rDNA, containing variable subregion V4, was used for molecular identification of studied isolates. For amplification of this region in Heterokontophyta species *Plagiotropis lepidoptera* var. *proboscidea* and *Gonyostomum semen*, we used a pair of primers—D512 for and D978rev (length 391–394 bp) from the work of Zimmermann et al. [15]. The part of nuclear SSU rDNA of the Dinoflagellata *Unruhdinium kevei* (length 923 bp) was amplified using primers EukA [16] and picoR2 [17]. Also, to improve the unclear phylogenetic position of *Plagiotropis lepidoptera* var. *proboscidea*, partial nucleotide sequences of the gene *rbc*L cpDNA were determined with primers rbcL66+ and rbcL1255—(length 800) from the publication Alverson et al. [18].

Amplification of all studied fragments was carried out using the premade mix ScreenMix (Evrogen, Moscow, Russia) for the polymerase chain reaction. The amplification conditions for all ribosomal genetic fragments were: an initial denaturation of 5 min at 95 °C, followed by 45 cycles at 94 °C for denaturation (30 s), 52 °C for annealing (30 s) and 72 °C for extension (50–90 s), and a final extension of 10 min at 72 °C. The conditions of amplification for the *rbc*L fragment were the same, with the exception of the annealing temperature—56 °C. The resulting amplicons were visualized by horizontal agarose gel electrophoresis (1.5%), and colored with SYBR Safe (Life Technologies, Carlsbad, CA, USA). Purification of DNA fragments was performed with the ExoSAP-IT kit (Affymetrix, Santa Clara, CA, USA) according to the manufacturer’s protocol. All fragments were decoded from two sides using forward and reverse PCR primers and the Big Dye system (Applied Biosystems, Foster City, CA, USA), followed by electrophoresis using a Genetic Analyzer 3500 sequencer (Applied Biosystems, Foster City, CA, USA).

### 2.4. Alignment and Phylogenetic Analysis

The received sequences were submitted to the GenBank database under the accession numbers OR936026, OR936027, OR936470-OR936474. The sequences were checked manually and assembled using MegaX [19]. The newly determined sequences were aligned to other sequences from the GenBank database. The sequences of *Gonyostomum semen* (Raphidophyceae) were supplemented by five other *Gonyostomum semen* strains and from other 18 representatives of the family Vacuolariaceae. Two strains of *Heterosigma akashiwo* (Y. Hada) Y. Hada ex Y. Hara & M. Chihara (Heterokontophyta, Chattonellaceae) were chosen as the out-group. The dataset for analysis of *Unruhdinium kevei* phylogenetical position consisted of 35 sequences of the family Kryptoperidiniaceae. Two species of the genus *Perkinsus* (class Perkinsea) were presented as the out-group. The assembling of datasets for the diatom *Plagiotropis lepidoptera* (Bacillariophyceae) was carried out using Diat. barcode v11.1 [20] to address the methodological problem of the dataset’s incongruence. Therefore, the final concatenated dataset 18S + *rbc*L contained 33 sequences of the order Naviculales from the barcode database. Also, 18S rDNA sequences of *Plagiolemma distortum* and *Plagiotropis* sp. (Bacillariophyceae) (MG587952–MG587955) [21] were added to the matrix and two strains of araphid diatom *Ulnaria ulna* (Nitzsch) Compère (Bacillariophyceae) were chosen as the out-group. The sequences of the ribosomal gene were aligned using global SILVA alignment in the SINA v1.2.11 [22]. The sequences of the cpDNA gene *rbc*L were aligned using MAFFT v7 with model E-INS-i [23]. Evolution model selection was performed in MegaX. Phylogenetic trees were constructed using the maximum likelihood (ML) method in the MegaX software (https://www.megasoftware.net/web_help_11/index.htm#t=Preface.htm). Bootstrap analyses with 1000 replicates were calculated to estimate statistical reliability. The Adobe Photoshop CC (19.0) program was used to edit trees.

## 3. Results

### 3.1. Environmental Indicators

The studied watercourses flow through the south of the taiga zone (60–70% forest) and have a swampy catchment area. The main source of river nutrition is snow cover (50–90% of the annual flow), rainfall is 2–15%, and groundwater is 15–25%. The underlying rocks of the Vetluga River catchment area mainly consist of carbonate clays with layers of marl and limestone. Those of the Kerzhenets and the Vishnya rivers are sands. The character of the catchment area determines the hydrochemical parameters of the rivers, the waters of which have low mineralization values, slightly acidic pH values, and increased chromaticity. The rivers are not affected by strong anthropogenic influence. The river catchment areas are partially located in the reserve area. The main hydrological and hydrochemical characteristics of the studied rivers are given in Table 2.

### 3.2. Phytoplankton Species Composition

In total, taxonomic diversity amounted to 812 species (901 species and intraspecific taxa), belonging to 254 genera, 36 orders, 15 classes, and 8 phyla, which were identified in the phytoplankton of the Vetluga, the Kerzhenets, and the Vishnya rivers. The basis of the algal flora (82.4%) was formed by representatives of 3 phyla: Chlorophyta, Ochrophyta (Bacillariophytina, Ochrophytina), and Euglenophyta.

The use of a variety of approaches (light and electron microscopy, molecular genetic studies) made it possible to identify 6 invasive species of algae in the phytoplankton of the Kerzhenets, the Vetluga, and the Vishnya rivers, most of which belong to the Bacillariophytina subphylum (*Thalassiosira incerta* I.V. Makarova, *T. faurii* (Gasse) Hasle, *Skeletonema subsalsum* (A. Cleve) Bethge, *Plagiotropis lepidoptera* var. *proboscidea*). Species from the phylum Dinoflagellata (*Unruhdinium kevei* (Grigorszky & F. Vasas) Gottschling) and the class Raphidophyceae (*Gonyostomum semen* (Ehrenberg) Diesing) were also detected.

### 3.3. Description, Distribution, and Ecological Characteristics of Invasive Species

#### 3.3.1. Invasive Raphydophytes

**Figure 2:**
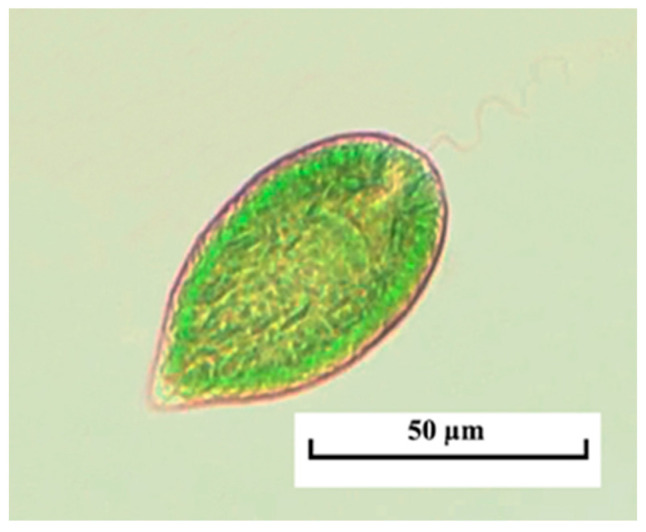
*Gonyostomum semen* in the plankton of the Kerzhenets River, light microscopy.

*Gonyostomum semen* is a typical inhabitant of boreal lakes; it has seriously expanded its range in European water bodies over the past few decades with a remarkable increase in quantitative development characteristics [24]. The first information about the appearance of *Gonyostomum semen* in the Kerzhenets River dates back to the mid-1980s when an outbreak of development of this species was noted (up to 6.0 g/m^3^) [25,26]. In the modern period (2000s to present), *G. semen* has been regularly found in summer phytoplankton communities of meso- and polyhumous waters of the Kerzhenets and the Vishnya rivers (Figure 2). Unlike the oxbow lakes of the mentioned rivers, where this representative could cause “blooming” (the contribution of this species to the total biomass was 98%, with absolute values of almost 100 g/m^3^), it was less abundant in the studied rivers. Thus, in the middle course of the Kerzhenets River, the abundance of *G. semen* varied from 0.001 to 0.005 × 10^6^ cells/L, biomass –from 0.02 to 0.132 g/m^3^, while its share in the overall indicators ranged from 1 to 6%. The degree of development of this species in the studied phytoplankton communities is probably underestimated due to the rapid destruction of cells in fixed material.

**Figure 3:**
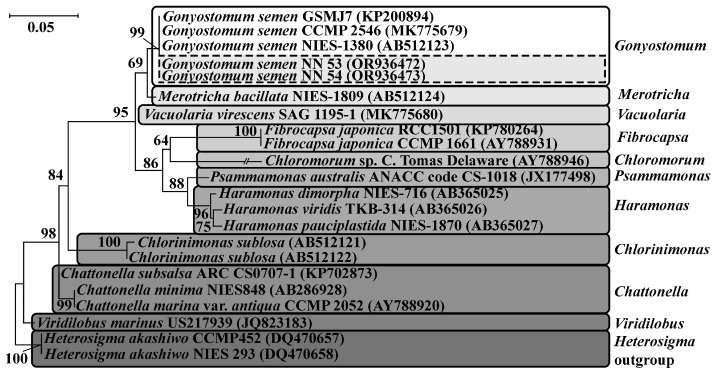
Maximum likelihood tree of the partial nuclear-encoded SSU rDNA data set of the family Vacuolariaceae. Bootstrap values > 50% are shown in the nodes of tree. Scale bar indicates substitution per site. Isolates of *Gonyostomum semen* from the Kerzhenets River are highlighted with a dotted rectangular.

Phylogenetic relationships inferred using ML from the dataset of nuclear-encoded SSU show that the isolates NN53 and NN54 form one clade with other *G. semen* strains collected around the world (GenBank accession numbers: KP200894, MK775679, AB512123) with high statistical support (ML = 99). This fact reliably confirms the finding of *G. semen* in the investigated river (Figure 3).

#### 3.3.2. Invasive Dinoflagellates

**Figure 4:**
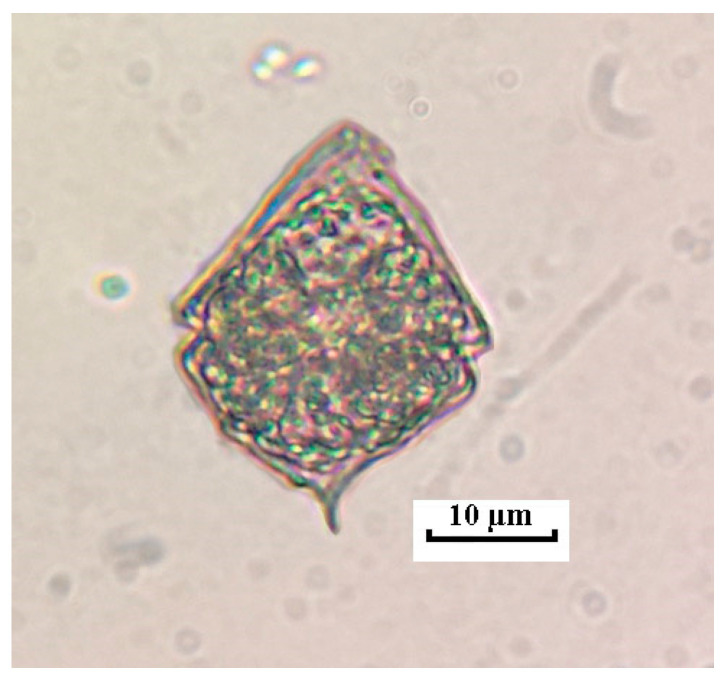
*Unruhdinium kevei* cell in the phytoplankton of the Kerzhenets River, light microscopy.

The first data about the appearance and naturalization of *Unruhdinium kevei* date back to 2006 in the Kerzhenets River, to 2010, after an abnormally hot summer in the Vetluga River. As for the Vishnya River, the first finding was made in 2016, where a few cells were observed in the composition of summer plankton (Figure 4).

Thus, *U. kevei* has diamond-shaped cells, oval, or highly compressed in cross sections. The girdle is descending, dividing the cell into two cone-shaped halves. A cell with a thick shell. The hypovalve is a little bit shorter than the epivalve; its first antapical plate has a spine, the length of which is from 3 to 5 μm. At the top of the epivalve, there is an apical pore, covered by the outgrowth of the fourth apical plate. Dimensions are: 31–47 µm long, 25–37 µm wide, 18–25 µm thick [27,28].

**Figure 5:**
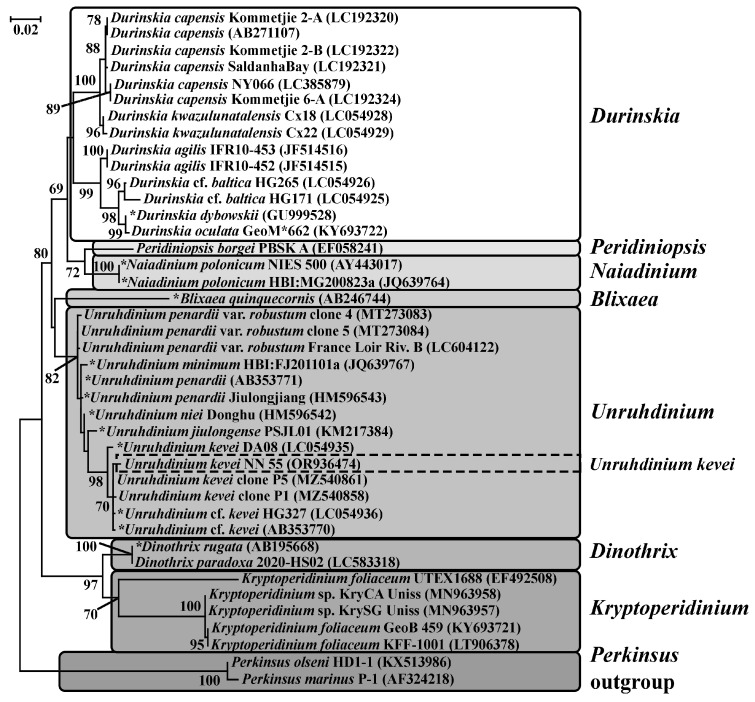
Maximum likelihood tree of the partial nuclear-encoded SSU rDNA data set of the family Kryptoperidiniaceae. Bootstrap values > 50% are shown in the nodes of the tree. The scale bar indicates substitution per site. Isolates of *Unruhdinium kevei* from the Kerzhenets River are highlighted with a dotted line. The discrepancies between the species name according to the taxonomy used in the present study and the species name in GenBank are marked with an asterisk.

Phylogenetic relationships inferred using ML from the dataset of nuclear-encoded SSU, show that the isolates NN55 formed one clade with other species of the genus *Unruhdinium*: *U. jiulongense* (H. Gu) Gottschling, *U. niei* (G.X. Liu & Z.Y. Hu) Gottschling, *U. penardii* (Lemmermann) Gottschling, *U. penardii* var. *robustum* (Qi Zhang, G.X. Liu & Z.Y. Hu) Gottschling, *U. minimum* (Qi Zhang, G.X. Liu & Z.Y. Hu) Gottschling with moderate support (ML = 81). Inside this clade, NN55 formed one subclade with *Unruhdinium kevei* isolates with a high level of support (ML = 98). All of the present members in this subclade have their origin in the fresh waters of Japan (AB353770, LC054935, LC054936) and South Korea (MZ540858, MZ540861) (Figure 5).

**Figure 6:**
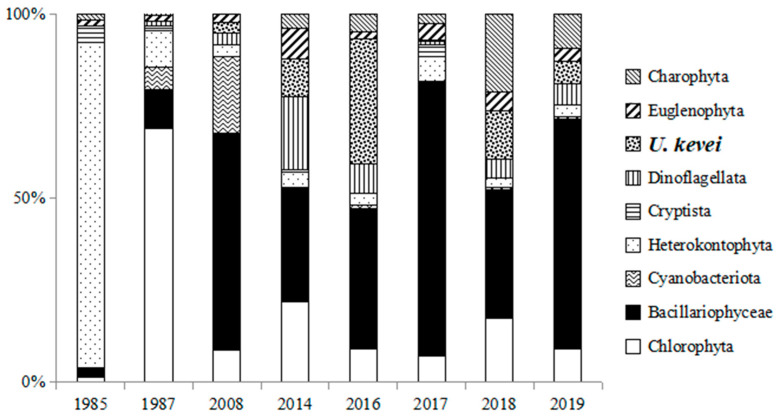
The contribution of different systematic groups and *Unruhdinium kevei* to the total biomass of phytoplankton in the Kerzhenets River in different years of research.

Since 2008, in the Kerzhenets River, an increase in the participation of dinophyte algae in the formation of phytoplankton communities (up to 1.86 g/m^3^—69% of the total biomass) (Figure 6) has been shown. Species of the genera *Gymnodinium* (up to 23% of the total biomass) and *Unruhdinium kevei* (up to 25% of the total biomass) were observed among the dominant dinoflagellates.

*U. kevei*, whose maximum development was registered at the end of July and throughout August 2008, together with other representatives of dinoflagellates, was part of the potamophytoplankton dominant species. At the same time, its abundance varied from 8 to 144 × 10^3^ cells/L, and its biomass from 0.09 (8% of the total biomass) to 0.7 g/m^3^ (34% of the total biomass). In 2014, the abundance of the species varied from 6 × 10^3^ cells/L to 120 × 10^3^ cells/L. The maximum development of *U. kevei* occurred in August, when its biomass reached a maximum value of 1.28 g/m^3^ (66.5% of the total biomass). In 2016, the development of this species was also noted with values of abundance ranging from 5 to 30 × 10^3^ cells/L and biomass—from 0.03 (5% of total biomass) to 0.58 g/m^3^ (55% of total biomass). In 2017, only single cells of *U. kevei* were found in phytoplankton, which is probably caused by the climatic features that determine the water regime of the studied rivers. In recent years, this species has also been a part of the dominant species complexes with quantitative development indicators ranging from 0.06 to 0.37 g/m^3^ (up to 31%) in 2018 and from 0.03 to 0.67 g/m^3^ (up to 31%) in 2019 (Table 3).

In the Vetluga River, in contrast to the Kerzhenets River, quantitative indicators of the development of *U. kevei* were characterized by lower values (Table 3). Only a few finds of it were noted in summer samples of 2010 and 2014. The maximum biomass value was detected in the summer of 2016 and amounted to 0.74 g/m^3^ (26% of total biomass).

In the Vishnya River, a gradual introduction of *U. kevei* into planktonic communities is currently being experienced along with the absence of records of significant development indicators, since only single cells were found in autumn samples. The maximum biomass values reached 0.01 g/m^3^ (3.5% of the total biomass) (Table 3).

In all of the studied rivers, the time range of occurrence of *U. kevei* covers the period from late spring (April–May) to early autumn (September). The development of this species showed a significant positive correlation with water temperature and a negative correlation with water level (Table 4).

#### 3.3.3. Invasive Diatoms

**Figure 7:**
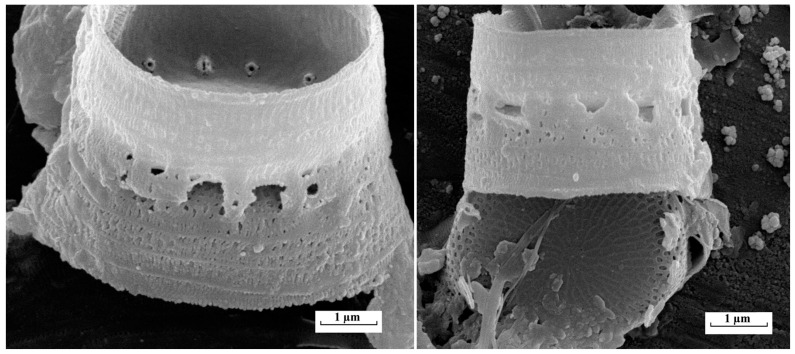
*Skeletonema subsalsum* in the plankton of the Vetluga River, scanning electron microscopy.

Gradual invasion of *Skeletonema subsalsum* into the reservoirs of the Volga River and the vegetation of this species was recorded in 1960–1970 [29]. In the Vetluga River, the species was revealed in the summer season of 2016, and its biomass reached 8% of the total (0.05 g/m^3^) with an average value of 0.04 ± 0.01 g/m^3^. In recent years, according to our data (unpublished), only sporadic findings of *S. subsalsum* have been recorded in planktonic water samples. Findings of *Skeletonema subsalsum* in planktonic samples were confirmed using light and electron microscopy (Figure 7).

**Figure 8:**
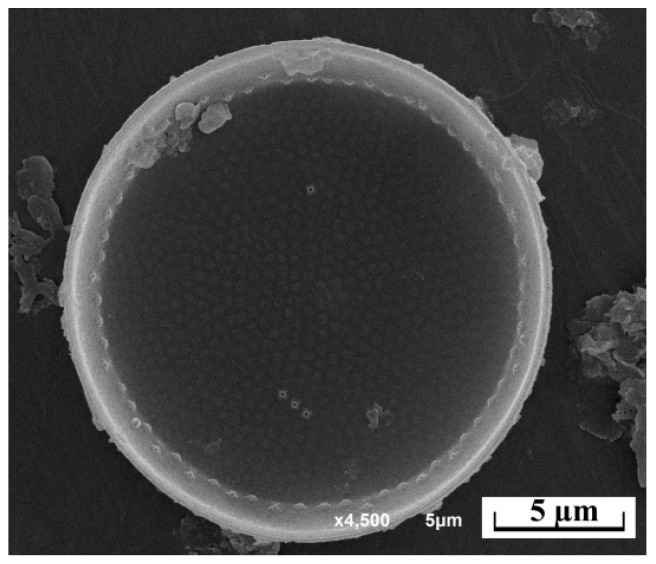
Invasive species *Thalassiosira faurii* in the phytoplankton of the Kerzhenets River, scanning electron microscopy.

The results of electron microscopy also made it possible to identify the centric diatom *Thalassiosira faurii* in the phytoplankton composition of the Kerzhenets River (Figure 8). The morphological features of the *T. faurii* frustule were described earlier in the work of Genkal and Korneva [30] when studying the reservoirs of the Lower Volga. Due to the complex structure of the frustule and the presence of a number of features visible only under electron microscopy (the presence of central and marginal outgrowths, bilabial outgrowths fultoportulas), identification of the species in a light microscope and its assignment as part of the algal community is not possible. 

**Figure 9:**
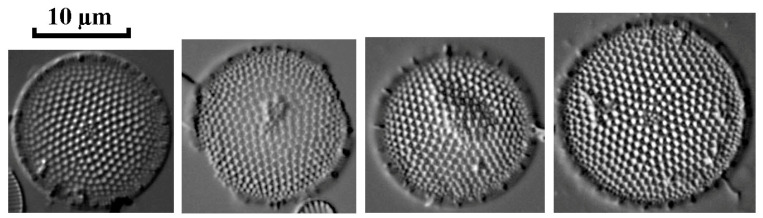
*Thalassiosira incerta* in the phytoplankton of the Kerzhenets River, light microscopy.

The results of a diatom analysis revealed the presence of the *Thalassiosira incerta* species in the Kerzhenets River (Figure 9). No significant developmental indicators were noted for this species since only single valves were found in permanent diatom microslides.

**Figure 10:**
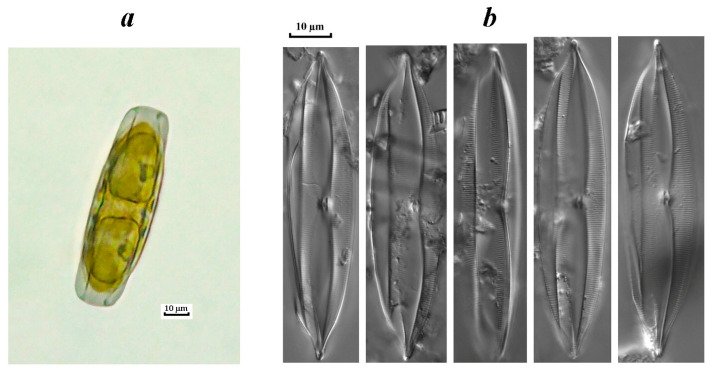
The invasive species *Plagiotropis lepidoptera* var. *proboscidea* in the phytoplankton of the Vetluga River: (**a**)—an organism taken from a planktonic sample (cell view from the girdle), (**b**)—a cleaned valve. Light microscopy.

The appearance and vegetation of a benthic species of the genus *Plagiotropis*, as an invasive component of phytoplankton communities, were noted in the studied rivers (the Vetluga and the Kerzhenets rivers) for the first time. A detailed study of the frustule structure allowed us to identify the found species as *Plagiotropis lepidiptera* var. *proboscidea* (Figure 10), which registered throughout the entire growing season (April–October), mainly during maximum water warming (R_s_ = 0.51, *p* ≤ 0.05).

**Figure 11:**
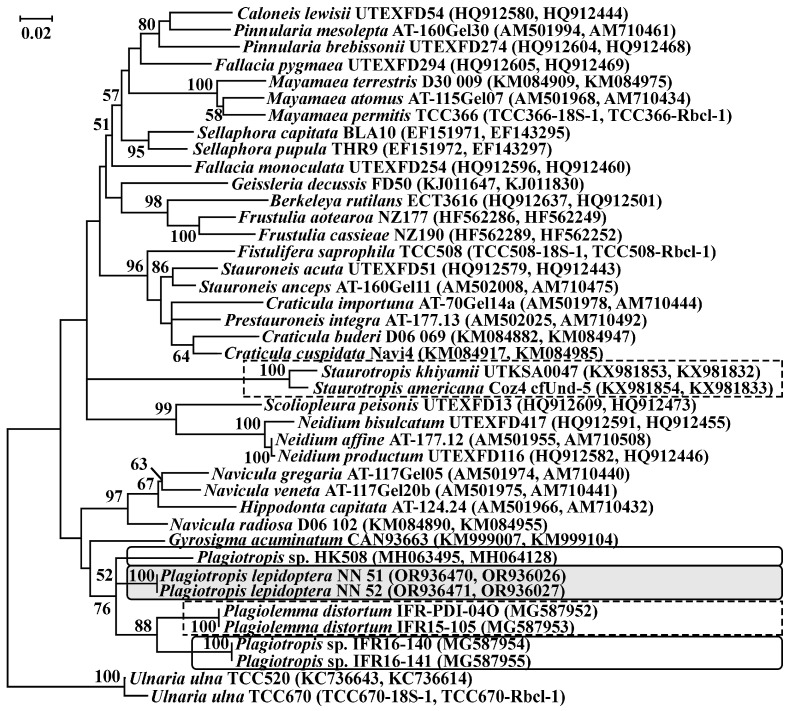
Maximum likelihood tree of the partial nuclear-encoded SSU rDNA and chloroplast *rbc*L cpDNA combined dataset of the order Naviculales. Bootstrap values > 50% are shown in the nodes of the tree. The scale bar indicates substitution per site. Isolates of *Plagiotropis lepidiptera* var. *proboscidea* from the Vetluga River are highlighted with a dotted darkened rectangle, other Plagiotropis isolates are marked with an oval, and other members of the family Plagiotropidaceae are in a solid rectangle.

Phylogenetic relationships inferred using the ML of a concatenated dataset of nuclear-encoded SSU rDNA and plastid-encoded *rbc*L cpDNA show that the isolates NN51 and NN52 formed an independent clade, sister to a branch of *Plagiotropis* sp. with low support (ML = 52) (Figure 11). The analyses of datasets of SSU rDNA and plastid-encoded *rbc*L cpDNA separately repeat the individual position of *Plagiotropis lepidoptera* var. *proboscidea* isolates with low bootstrap support (ML = 56 for SSU rDNA analysis).

In the Vetluga River, in different years of research, *Plagiotropis* was part of the dominant complexes of the river, along with other diatoms in the second half of the summer season. The maximum abundance and biomass registered in 2014 were 20 × 10^3^ cells/L (4.7% of the total abundance) and 0.43 g/m^3^ (34.3% of the total biomass), and in 2016, they were 40 × 10^3^ cells/L (0.3% of the total abundance) and 1.2 g/m^3^ (42.4% of the total biomass), respectively. As for the Kerzhenets River, only single findings of *P. lepidoptera* var. *proboscidea* were made (Table 5).

## 4. Discussion

Globally, the spread of alien species is considered to be one of the most important threats to natural biodiversity [31]. Thus, the noticeable influence of alien species of higher aquatic plants, algae, and cyanobacteria on the ecological state of freshwater bodies was shown in a number of works [2,3,32,33].

The negative impact of invasive species on biodiversity is also revealed as changes in the genetic diversity of species and in the complete disappearance of native species. As a result, the restructuring of the entire ecosystem is visible [34]. Factors such as climate change, degradation, and pollution of natural habitats increase the negative impacts of biological invasions on natural ecosystems [35,36]. The intensity of these processes in freshwater ecosystems is higher than in terrestrial or marine ones [37].

In our studies, we detected some trends in the changes in the hydrochemical composition of the waters in the Volga River tributaries. The studied rivers flow within a swampy catchment area; therefore, they have low water mineralization values (52–150 mg/L) and have slightly acidic or neutral pH values (6–7.8) with a pronounced gradient of water chromaticity. In a 50-year series of observations, a noticeable enlargement in chromaticity and total iron content was established in the studied rivers in a long-term aspect. So, chromaticity values were: in the Vetluga River—W = 2.59, *p* = 0.009 and in the Kerzhenets—W = 2.39, *p* = 0.016; and total iron content—W = 2.02, *p* = 0.043; W = 2.20, *p* = 0.027, respectively. Against the background of a certain growth in COD values in the water of both rivers, the share of organic matter easily oxidized by bacteria (according to BOD5) in the total mass of organic matter in the Kerzhenets River has increased slightly over 50 years, and in the Vetluga River, this parameter decreased by more than 2 times (W = 2.42, *p* = 0.015) [12].

Our long-term research demonstrated the rich species composition of phytoplankton in the studied rivers. It was noted that there were no significant changes in the taxonomic composition. The main changes concerned transformations in dominant species composition, including the expansion and naturalization of alien species. Changes in the structural and functional characteristics of algal communities and changes in the speed and direction of phytoplankton communities succession were also recorded for other water bodies of the Volga River basin [1,8,12,38,39,40,41].

It is currently assumed that the main reasons for the expansion of alien algae species in the Volga River basin are hydraulic construction and global climate change, which subsequently led to a transformation of the hydrological and hydrochemical regimes, changes in water temperature, transparency, chromaticity, and flows of nutrients and minerals [1,8]. Information regarding the ionic composition of waters where the appearance of invasive species is observed is extremely insufficient. More attention is paid to the content of nutrients, which is necessary to estimate the trophic status of a water object. However, according to Korneva [1] and Padisak and Reynolds [42], changes in the concentration and share of salts can serve as a trigger for invasive species expansion.

Two periods were distinguished in the spread of invasive algae species in the Volga River basin: the 1960s and 1980s. The first period is connected with the finishing of the construction of the Volga-Kama cascade reservoirs and the appearance of the first invader species *Skeletonema subsalsum* (A. Cleve) Bethge and representatives of the genus *Thalassiosira* with their subsequent distribution. The second period is associated with the filling of the last reservoir in the Cheboksary cascade (1981) and the invasion of the diatom algae *Actinocyclus normanii* (Greg.) Hust [8,43].

Invasive algae species representatives from Cyanophyceae are noted within the Volga basin: *Raphidiopsis raciborskii* (Woloszynska) Aguilera, Berrendero Gómez, Kaštovský, Echenique & Salerno (the Kuibyshev reservoir and the Lower Volga), *Chrysosporum bergii* (Ostenfeld) Zapomelová, Skácelová, Pumann, Kopp & Janecek (reservoirs of the Middle and the Lower Volga). The origin of these cyanobacteria belongs to tropical and subtropical latitudes, they are gradually spread into the water bodies of Western Europe, from where they subsequently penetrate the boreal region of the temperate zone. Their noticeable development is associated with eutrophic stratified water bodies. Among them, there are species that cause or potentially can cause the “blooming” of water [44] and synthesize cyanotoxins [1].

Raphidophyte algae *Gonyostomum semen* (Ehr.) Diesing which penetrated the territory of the former USSR from Europe, has recently become widespread. Being a typical inhabitant of boreal lakes, *G. semen* has seriously expanded its range in European water bodies over the past few decades with an increase in abundance [24]. It develops more intensively in well-warmed stratified lakes with high values of water chromaticity [1]. Massive vegetation of this species was recorded in Russia (1985) in small low-mineralized rivers with high chromaticity values in the left bank area of the Nizhny Novgorod region [25]. A number of publications have shown that increasing average annual temperature and, as a consequence, the longer duration of the growing season, higher values of water chromaticity, phosphorus, and total organic carbon content are the main factors determining the high spreading rates of this species [24,45,46].

*Unruhdinium kevei*, as a representative of the freshwater dinoflagellates, has been widespread recently. This species was first described as being from the tributaries of the Tisa River, Hungary, according to 1986–1995 data. During this period of time, the species was recorded in many European countries [47]. According to L.G. Korneva, *U. kevei* was first discovered in 1989 in the Mologa and Vochkoma rivers, flowing into the Molozhsky reach of the Rybinsk reservoir, and also in the coastal shallow waters of the Molozhsky reach [1]. The morphology and size characteristics of *U. kevei* from the left bank tributaries of the Cheboksary Reservoir generally correspond to those given in the literature [27,28].

Literary data [1,40,43,47] and the results of our own observations demonstrated that *U. kevei* is distributed in rivers of large and small size and reservoirs of various trophic statuses (from oligo- to eutrophic), in a fairly wide range of concentrations of total nitrogen and phosphorus, but is quite demanding on the temperature and alkalinity of the water. The highest values of development were observed in summer or early autumn at a water temperature of 15–26 °C, which could cause the “blooming” of water. Also, high values of species development were noted in bird nesting areas, where the biomass of *U. kevei* reached 2.44 g/m^3^ [1,47,48,49].

Such algae species as *Skeletonema subsalsum*, *Actinocyclus normanii*, *Thalassiosira incerta*, *Thalassiosira lacustris* (Grun.) Hasle, *Thalassiosira faurii*, *T. pseudonana* Hasle et Heimdal., *T. weissflogii* (Grun.) Fryxell et Hasle), *T. proschkinae* Makar. [1] were registered in the list of invasive species of diatoms in the Volga River basin before our research (in all reservoirs of the Volga cascade) [50]. Currently, about eight species of the genus *Thalassiosira* are recorded in the phytoplankton communities of the Volga River, but only *T. incerta* can vegetate in large quantities. This species was noted as a part of the dominant planktonic complex downstream of the Oka River in 2011 [51].

In the phytoplankton of the studied rivers, four species of diatoms were classified as invasive among more than 308 species of varieties and forms of Bacillariophyta phylum noted there. These invasive species are representatives of marine and brackish water ecosystems. Gradual invasion of *Skeletonema subsalsum* into the reservoirs of the Volga River and the vegetation of this species there was recorded in 1960–1970 [29]. This heat-preferring species developed in the Gorky Reservoir with optimum temperature conditions of 16–22 °C. The building of the Cheboksary reservoir contributed to the growth of this species’ abundance (up to 0.74 ± 0.04) and maximum biomass values (up to 2–10 g/m^3^ at some stations) [52].

Generally, the abundance of most species of the genus *Thalassiosira* in phytoplankton water samples did not exceed values 50–100 × 10^3^ cells/L, and only *T. incerta* reached the values of quantitative development compared with ones specific for eutrophic and hypertrophic rivers [52]. This species has successfully settled down in the waters of the Cheboksary Reservoir (abundance values were up to 444 × 10^3^ cells/L, biomass—up to 1.5 g/m^3^), but the maximum development rates were noted in the mouth area of the Oka River (10.5 × 10^6^ cells/L, 23.4 g/m^3^) [51]. Currently, this species is a permanent component of phytoplankton communities in the Oka River and other large tributaries of the Volga Rivers. Other species of this genus were recorded sporadically or their abundance did not exceed 100 × 10^3^ cells/L.

The listed representatives of invasive flora are components of plankton communities. Findings of benthic species were less common. In particular, there is information about the presence of a benthic species in the Lower Volga—*Halamphora coffeiformis* (C. Agardh) Mereschkowsky (Bacillariophyceae) [39]. In our work, *Plagiotropis lepidoptera* var. *proboscidea* was recorded in the Middle Volga basin for the first time.

Combinations of modern research approaches aimed at studying the biodiversity of algae, protists, and prokaryotic organisms more and more often reveal the hidden, undescribed diversity of these organisms. Part of this diversity is presented by complex cultured or uncultured organisms. Single-cell PCR is one of the possible prospective approaches to studying this phenomenon. In our research, we confirmed the identification with molecular data of the raphidophyte species *Gonyostomum semen*, which is consistent with the data of Lebret et al. [53]. The dinophyte species *Unruhdinium kevei* is also confirmed by molecular data in the Kerzhenets River. Due to the problem of cultivation of this organism, there are a small number of sequences in the nucleotide databases. The sequences originated only from four localities, which refer to the freshwater bodies of the Asian region (Japan and South Korea). The distribution in inland waters of other regions remains unstudied.

In contrast to the species discussed above, the phylogenetic position of *Plagiotropis lepidoptera* var. *proboscidea* was not clear. In all phylogenetic analyses, the isolates NN51 and NN52 situate without high bootstrap support among members of the family Plagiotropidaceae, but they do not form a reliable cluster with any of them. Three other unidentified species of the genus *Plagiotropis* originated from marine habitats, strain HK508 is from Condado Lagoon, Puerto Rico, and isolates IFR16-140 and IFR16-141 originate from Meyran Lagoon, France. Other genera of the family Plagiotropidaceae, *Plagiolemma* and *Staurotropis* do not have a constant origin location, it changes depending on dataset analysis. Therefore, these facts point to the polyphyletic status of both genus *Plagiotropis* and family Plagiotropidaceae, their further revision is needed. The genus *Plagiotropis* and its family are poor in species, which, in many cases, have been studied on the basis of valve morphology and described in the last century. Despite this, additions to the analyses of new species or increasing the number of nucleotide sequences of studied species can improve the resolution of future phylogenetic studies.

According to the literature, species of the genus *Plagiotropis* are often found in phytoplankton communities of marine [54], brackish-water, and highly mineralized [55] water bodies, predominantly developing in microphytobenthos. Currently, *Plagiotropis lepidoptera* var. *proboscidea* is considered an invader species of the Caspian Sea [56], which is probably debatable, since findings of the species were recorded in the Caspian Sea back in 1938 [57]. Proshkina-Lavrenko [58] noted that *P. lepidoptera* var. *proboscidea* is often found along the entire coast of Europe from the North Sea to the Mediterranean. The uniqueness of this find is revealed by the fact that the species, usually indicated for the flora of reservoirs with medium and high salinity values, up to marine conditions, was found in low-mineralized humic rivers. This determines the relevance of further study of the invasive algae genetic diversity in the Volga River basin and the formation of their adaptive mechanisms of resistance to salinity.

## 5. Conclusions

The spread and subsequent development of invasive algae species with a predominance of Bacillariophyta representatives probably indicate a change in the hydrological and climatic regime, trophic status, and mineralization of waters. Findings of *Unruhdinium kevei* (all the studied rivers), *Plagiotropis lepidoptera* var. *proboscidea* (the Kerzhenets and the Vetluga rivers), *Thalassiosira faurii*, *T. incerta* (the Kerzhenets River), *Skeletonema subsalsum* (the Vetluga River), *Gonyostomum semen* (the Kerzhenets River) demonstrate active processes of alien algal species expansion into the tributaries of the Cheboksary reservoir. Information on the distribution of *U. kevei*, *T. incerta*, *S. subsalsum*, and *G. semen* in European rivers and the Volga reservoirs has been presented in the literature since the mid-20th century. Data on the development of *P. lepidoptera* var. *proboscidea* represent the first findings of benthic phytoplankton community species belonging to marine and highly mineralized waters in the reservoirs of the river Volga basin.

Analysis of seasonal and inter-annual phytoplankton development showed that *Unruhdinium kevei* and *Plagiotropis lepidoptera* var. *proboscidea* are capable of actively developing and transforming the structure of aboriginal communities as a part of dominant complexes. Probably, the climatic anomalies of 2010 served as a trigger for the rapid development of these species in the planktonic and benthic communities of the studied rivers. *Unruhdinium kevei* is found in all rivers, characterized by different values of water chromaticity, pH, mineralization, and trophic status, which indicates its wide ecological adaptability. The highest rates of development of *U. kevei* were registered in the Kerzhenets river (up to 66.5% of the total biomass) during the summer low-water period, with maximum warming of the waters and a low water level. Information on the ecology of *Plagiotropis lepidoptera* var. *proboscidea* is not numerous in the literature. It is known that species of the genus *Plagiotropis* are only known to take part in the formation of microphytobenthos communities in marine, brackish, and highly mineralized water bodies [59].

Molecular approaches for invasive species identification proved its relevance since most of these species are difficult to cultivate and can quickly degrade in fixed material. The use of molecular approaches for invasive species identification has shown its relevance since most of these species are difficult to cultivate and can quickly degrade in fixed material. Reliable information about the further role and ecology of identified alien species can be obtained by organizing long-term ecological and floristic monitoring of different water bodies especially ones on the territories of nature reserves. Our research results determine the importance of predicting changes under conditions of anthropogenic influence and climate change.

## Figures and Tables

**Table 1:** Some features of sample stations.

Station	River	GPS Coordinates	Collection Years
1	Vetluga	57.39 N, 45.10 E	2010
2	Vetluga	57.12 N, 45.18 E	1987
3	Vetluga	56.85 N, 45.43 E	2014, 2016, 2018
4	Vetluga	56.32 N, 46.36 E	1972–1979
5	Kerzhenets	56.64 N, 44.67 E	1987
6	Kerzhenets	56.58 N, 44.65 E	1985
7	Kerzhenets	56.49 N, 44.79 E	2008, 2014, 2016, 2018, 2019
8	Kerzhenets	56.08 N, 44.97 E	1972–1979, 1981–1990
9	Vishnya	56.50 N, 44.81 E	2002, 2016

**Table 2:** The main hydrological and hydrochemical characteristics of the studied rivers (according to 2021 year of study).

Parameters	Vetluga	Kerzhenets	Vishnya
Length, km	889	290	27
Catchment area, km^2^	39,400	6140	250
Chromaticity, Pt-Co	92.8	96.5	207.13
pH	7.9	7.09	6.54
Mineralization, mg/L	145	100.7	75.3
HCO_3_^−^, mg/L	88.6	61.2	42.1
SO_4_^2−^, mg/L	8.02	7.4	4.0
Cl^−^, mg/L	5.6	4.8	1.0
Ca^2+^ + Mg^2+^, mg/L	27	21.1	17.5
Fe_total._, mg/L	0.22	0.38	0.45
P_total_, mg/L	35	55	63
N_min._, mgN/L	0.45	0.5	0.82
O_2_, mg/L	12.5	9.6	6.63
COD, mg O_2_/L	32.1	32.7	42.6
BOD_5_, mg O_2_/L	0.55	0.77	1.36

**Table 3:** Abundance (N, 10^3^ cells/L), share of the total abundance (N/N total, %), biomass (B, g/m^3^), and share of the total biomass (B/B total, %) of *Unruhdinium kevei* in the phytoplankton of the studied rivers.

Water Body, the Year of Sampling	N, 10^3^ cells/L	N/N Total, %	B, g/m^3^	B/B Total, %
Vetluga, 2010	0.02	0.31	0.44	32.8
Vetluga, 2014	0.02	2	0.31	12.4
Vetluga, 2016	0.021 ± 0.01	1	0.31 ± 0.11	29.7
Kerzhenets, 2008	0.07 ± 0.06	1.1	0.39 ± 0.3	15.1
Kerzhenets, 2014	0.048 ± 0.01	1.1	0.46 ± 0.11	26.2
Kerzhenets, 2016	0.04 ± 0.01	1.1	0.56 ± 0.15	39.7
Kerzhenets, 2017	0.01	0.5	0.057	2.1
Kerzhenets, 2018	0.02 ± 0.01	0.46	0.30 ± 0.07	24.8
Kerzhenets, 2019	0.02 ± 0.01	0.7	0.22 ± 0.06	12.4
Vishnya, 2016	0.001	0.5	0.01	3.5

**Table 4:** Significant (*p* ≤ 0.05) Spearman correlation coefficients of *U. kevei* development indicators and some environmental factors for the Kerzhenets River.

Parameters	Water Level	T, °C	pH	Conductivity	Chromaticity
*U. kevei* biomass	−0.79	0.59	0.75	0.61	0.64
*U. kevei* share in total biomass	−0.83	0.52	0.75	0.59	0.61

**Table 5:** Abundance (N, 10^6^ cells/L), share of the total abundance (N/N total, %), biomass (B, g/m^3^), and share of the total biomass (B/B total, %) of *Plagiotropis lepidoptera* var. *proboscidea* in the phytoplankton of the studied rivers.

Water Object, the Year of Investigation	N, 10^6^ cells/L	N/N Total, %	B, g/m^3^	B/B Total, %
Vetluga, 2014	0.01 ± 0.01	0.2	0.22 ± 0.10	14.6
Vetluga, 2016	0.02 ± 0.01	0.4	0.46 ± 0.19	44.1
Kerzhenets, 2019	0.004	0.3	0.069	6.4

## Data Availability

Data are contained within the article.
